# Phenylalanine Hydroxylase from *Legionella pneumophila* Is a Thermostable Enzyme with a Major Functional Role in Pyomelanin Synthesis

**DOI:** 10.1371/journal.pone.0046209

**Published:** 2012-09-26

**Authors:** Marte I. Flydal, Christa H. Chatfield, Huaixin Zheng, Felizza F. Gunderson, Oscar Aubi, Nicholas P. Cianciotto, Aurora Martinez

**Affiliations:** 1 Department of Biomedicine, University of Bergen, Bergen, Norway; 2 Department of Microbiology-Immunology, Northwestern University Medical School, Chicago, Illinois, United States of America; University of São Paulo, Brazil

## Abstract

**Background:**

*Legionella pneumophila* is a pathogenic bacterium that can cause Legionnaires’ disease and other non-pneumonic infections in humans. This bacterium produces a pyomelanin pigment, a potential virulence factor with ferric reductase activity. In this work, we have investigated the role of phenylalanine hydroxylase from *L. pneumophila* (lpPAH), the product of the *phhA* gene, in the synthesis of the pyomelanin pigment and the growth of the bacterium in defined compositions.

**Methodology/Principal Findings:**

Comparative studies of wild-type and *phhA* mutant corroborate that lpPAH provides the excess tyrosine for pigment synthesis. *phhA* and *letA* (*gacA*) appear transcriptionally linked when bacteria were grown in buffered yeast extract medium at 37°C. *phhA* is expressed in *L. pneumophila* growing in macrophages. We also cloned and characterized lpPAH, which showed many characteristics of other PAHs studied so far, including Fe(II) requirement for activity. However, it also showed many particular properties such as dimerization, a high conformational thermal stability, with a midpoint denaturation temperature (*T*_m_) = 79±0.5°C, a high specific activity at 37°C (10.2±0.3 µmol L-Tyr/mg/min) and low affinity for the substrate (*K*_m_ (L-Phe) = 735±50 µM.

**Conclusions/Significance:**

lpPAH has a major functional role in the synthesis of pyomelanin and promotes growth in low-tyrosine media. The high thermal stability of lpPAH might reflect the adaptation of the enzyme to withstand relatively high survival temperatures.

## Introduction

The genus *Legionella* is constituted by gram-negative motile rods. Presently 58 species are identified [1], [2], and many of these, including *Legionella pneumophila*, are reported to be pathogenic for humans. They exist naturally in freshwater habitats as parasites of protozoa and/or in biofilms, and while *L. pneumophila* thrives in temperatures between 25 and 45°C, it lives at 4–63°C within a pH range of 5.4–8.1 [3]. When standing water is aerosolized, primarily by human-made aquatic installations, *L. pneumophila* can infect a human host [4]. Once inside the lungs, it occupies alveolar macrophages and, if allowed to multiply and kill the macrophages, causes Legionnaires’ disease, a severe form of pneumoniae [5], [6]. However, the immune response of a healthy host is normally capable of eradicating the pathogen, making legionellosis largely a disease of immunosuppressed, elderly and smokers [7]. One of the mechanisms by which the innate immune system eradicates *L. pneumophila* is by iron-restriction in activated macrophages [8]. Because of its requirement for iron, *L. pneumophila* is grown on iron-supplemented media in the laboratory.

The genome of *L. pneumophila* includes a phenylalanine hydroxylase (PAH) ortholog, which is the product of the *phhA* gene. In other organisms, PAH catalyses the first step in the catabolic degradation of L-Phe using non-heme iron as cofactor and molecular oxygen and (6R)-L-*erythro*-5,6,7,8-tetrahydrobiopterin (BH_4_) as co-substrates [9], [10]. Traditionally, BH_4_ is also referred to as a cofactor. In bacteria, the PAH reaction is the initial step in a peripheral pathway where homogentisic acid (HGA) is a central intermediate [11]. The frequency of PAH in bacteria is not well established and its function has only been studied to some extent in *Pseudomonas aeruginosa* and *Pseudomonas putida* [12], [13]. In these bacteria the role of PAH is in the catabolic breakdown of L-phenylalanine as a source of carbon and nitrogen, respectively [13]. HGA is also the monomeric precursor of pyomelanin, which is a red-brown pigment produced after accumulation, excretion, auto-oxidation and self-polymerization of HGA [14]. Reduction or abolishment of homogentisate-1,2-dioxygenase (HmgA) activity leads to increased accumulation of HGA, and increased pyomelanin production [15], [16]. An equivalent of this situation occurs in alkaptonuria, a human genetic disease associated to mutations in *HmgA* [17]. In bacteria, pyomelanin is associated to a number of functions including anti-oxidation, virulence in pathogenic bacteria and assimilation of iron, among others [18], [19].

While mammalian PAH is the focus of intense investigation due to its implication in the disease phenylketonuria, a paradigm of inborn errors of metabolism [20], relatively few studies have been dedicated to the characterization of the function and importance of this enzyme in bacteria. PAH from *P. aeruginosa* [12], *Chromobacterium violaceum* (cvPAH) [21], [22], *Colwellia psychrerythraea 34H* (cpPAH) [23] and *Chloroflexus aurantiacus* (caPAH) [24] have been previously isolated and characterized, and for cvPAH and cpPAH their crystal structures have also been determined [22], [23]. The so-far characterized bacterial PAHs are monomeric, and adopt the same fold as the catalytic domain of mammalian PAHs [22], [23]. Also, similar to PAH from higher organisms, the bacterial enzymes are dependent on ferrous iron, located at the active site, for catalysis. Iron is also an important cofactor in other enzymes, and mechanisms for scavenging iron and limiting its availability have been developed in the pathogen and host, respectively (reviewed in [25]). Iron appears to be absolutely required for intracellular growth of *L. pneumophila* [8] and the bacterium has developed several strategies for iron acquisition (reviewed in [26]). One of the strategies seems to be via its pyomelanin as it was discovered that it has ferric reductase properties [19]. Thus, one important role of pyomelanin in *L. pneumophila* is reduction and acquisition of environmental iron.

**Figure 1:**
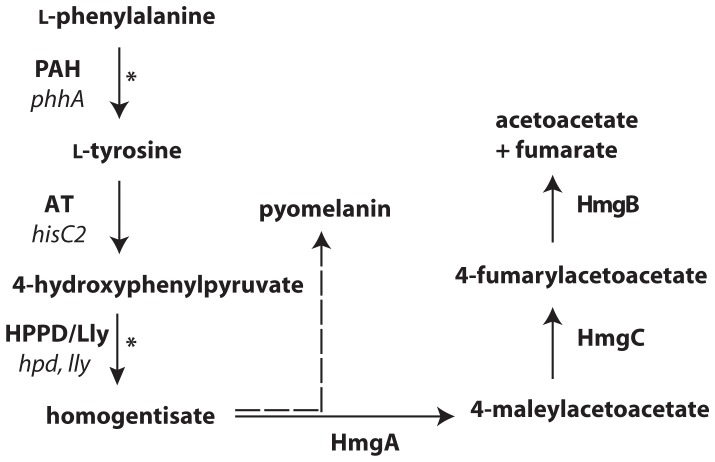
Pathway for phenylalanine/tyrosine catabolism and pyomelanin synthesis in *L. pneumophila.* The stippled arrow illustrates the polymerization of autoxidated homogentisate to make pyomelanin. PAH, phenylalanine hydroxylase; AT, amino acid transferase; HPPD/Lly, 4-hydroxyphenylpyruvate dioxygenase/legiolysin; HmgA, homogentisate-1,2-dioxygenase; HmgC, maleylacetoacetate isomerase; HmgB, fumarylacetoacetate hydrolase. The two *L. pneumophila* mutants used in this work are marked by asterisks and their gene names are shown (in italics).

So far most studies of pyomelanin-producing bacteria have focused on tyrosine as the substrate for HGA, and mutational studies aiming to investigate the regulation of pyomelanin synthesis have focused on enzymes in the latter part of its synthesis pathway (Fig. 1). In *L. pneumophila* both a pigment overexpressor (*hmgA*-) [19] and two pigment deficient strains (*pig*- and *lly*-) [27], [28] have been characterized. With the aim to elucidate the role of PAH from *L. pneumophila* 130b (lpPAH) in pyomelanin production we now constructed a *phhA* mutant which was comparatively characterized with the wild-type (WT) 130b in defined medium compositions. The transcription of *phhA* was assessed in different kinds of standard cultures and at 3 temperatures, and *phhA* linkage to *letA* was demonstrated. In addition, *phhA* was shown to be expressed in *L. pneumophila* growing in human macrophages. We also performed the cloning of the *phhA* gene and purified and characterized the expressed and purified lpPAH. Our results demonstrated that lpPAH promotes both growth in low-tyrosine media and the production of pyomelanin pigment by *L. pneumophila*, and the *phhA* mutant does not reach normal values of pigment production even upon supplementation with Tyr. Interestingly, lpPAH is dimeric and thermostable, with a melting point well above the optimal growth temperature of *L. pneumophila*. Moreover, it shows low affinity for L-Phe and its activity is dependent on the concentration of iron.

## Materials and Methods

### Bacterial Strains

*L. pneumophila* 130b (ATCC strain BAA-74, also known as AA100) served as our WT strain [19]. This serogroup-1 strain is a virulent clinical isolate. *Escherichia coli* DH5α was used as host for most of the recombinant plasmids generated in this study (Invitrogen, Carlsbad, CA). *E. coli* strain BL21 (Stratagene) was used for overproduction and purification of *L. pneumophila* PAH.

### Bacteriological Media and Assessments of Growth and Pigment Production

*L. pneumophila* strains were routinely cultured at 37°C on buffered charcoal yeast extract (BCYE) agar [29]. When selecting mutants, the BCYE agar was supplemented with kanamycin at 25 mg/ml, gentamicin at 2.5 mg/ml or chloramphenicol at 6 mg/ml. *E. coli* were grown in Luria-Bertani media, with kanamycin (50 mg/ml), gentamicin (2.5 mg/ml), chloramphenicol (30 mg/ml), or ampicillin (100 mg/ml). To monitor the basic extracellular growth capacity of *L. pneumophila*, bacteria grown on BCYE agar were inoculated into buffered yeast extract (BYE) or chemically-defined medium (CDM) broth, and then at various times post-inoculation, the optical density of the cultures was determined at 660 nm (OD_660_) [19]. To monitor the presence of secreted pigment, bacteria were inoculated into 30 ml of CDM containing different amounts of added tyrosine and then, after various periods of incubation, filter-sterilized culture supernatants were tested for their absorbance at 400 nm, as previously described [19].

### Mutant Construction and Complementation

As a first step toward obtaining a *phhA* mutant, the *phhA* gene was amplified by PCR from the genomic DNA of strain 130b using primers phhA-F (5′-ATGCAAGCTTATAATATCATGGTGTTCCGTCAGG-3′) and phhA-R (5′-ATGCGAGCTCGGACAATAAATCAAAGGGGGAATC-3′, and then the resulting fragment was cloned into the *Hind*III and *Sac*I restriction sites of pUC18. The resulting plasmid, pUCphhA, was digested with *Bse*RI, treated with Klenow fragment, and ligated to a *Hinc*II fragment of pMB2190 which carries a kanamycin-resistance (Km^r^) gene [30]. The result was a plasmid (pUCphhAKan) carrying *phhA* with a Km^r^ insertion at nt 162 of the gene’s coding region. pUCphhAKan was introduced into *L. pneumophila* 130b by transformation [31], and transformants were selected on antibiotic-containing BCYE agar. Insertion of the Km^r^ cassette into the chromosomal *phhA* gene was confirmed by PCR using primers phhA-F and phhA-R. Two independently derived *phhA* mutants were designated as NU406 and NU407. In order to perform *trans*-complementation of a *phhA* mutant, *phhA* was first cloned into pGEM-T Easy (Promega, Madison, WI) on a PCR product amplified from 130b using primers 5′-GGTACCCGTTGACTTTAATAGGCTGACCCCA-3′ and 5′-TCTAGATTCTAATCCACAAGTCCAGCTGTCTTAC-3′. After digestion of this resulting plasmid with *Kpn*I and *Xba*I, the *phhA*-containing fragment was ligated into pMMB2002 [30], placing *phhA* under control of the pTac promoter on the vector. The resulting plasmid, pPhhA, was electroporated [32] into mutant NU406, and colonies obtained on chloramphenicol-containing BCYE agar were confirmed by PCR to contain the intact *phhA* gene. In order to construct a *lly* mutant, the *lly* (*hpd*) gene was amplified by PCR from the genomic DNA of strain 130b using primers llyLpgFBcl1 (5′-TGATCCGAATGATCAGAGTGGA-3′, with a *Bcl*I restriction site underlined) and llyLpgXho1 (5′-ATGCGACTCGAGGAACGCAT-3′, with a *Xho*I restriction site underlined), and the resulting fragment was cloned into pGEM-T Easy (Promega, Madison, WI) to give plasmid pGlly. The *lly*-containing *Eco*R1 fragment of pGlly was then cloned into *EcoRI*-linearized pUC18 to give pUClly. Next, a gentamicin-resistance cassette was cloned into the *Nco*I site of pUClly inactivating the *lly* gene and resulting in pUClly:GNT. pUClly:GNT was introduced into *L. pneumophila* 130b by transformation [31], and transformants were selected on antibiotic-containing BCYE agar. Insertion of the gentamicin-resistance cassette into the chromosomal *lly* gene was confirmed by PCR using primers llyLpgFBcl1 and llyLpgXho1. The *lly* mutant of strain 130b was designated as NU408.

### DNA Isolation, PCR, and DNA Sequencing

DNA was obtained from *L. pneumophila* as described previously [33], and plasmids were routinely isolated from *E. coli* using the Plasmid Mini Prep kit (Bio Rad, Hercules, CA). All other DNA manipulations were performed using standard protocols [34]. Oligonucleotide primers for sequencing or PCR were synthesized at Integrated DNA Technology (Coralville, IA). Standard PCR was performed using Platinum Taq polymerase high fidelity (Invitrogen). DNA sequencing was done at the Northwestern Biotech Facility.

### RT-PCR Analysis of *L. pneumophila* Gene Expression

Standard reverse transcription (RT)-PCR was performed as previously described [19]. RNA was isolated from cultures of *L. pneumophila* in BYE and CDM media as well as growing in U937 macrophages (infected as described [35], [36]) using the RNA STAT-60 reagent according to the manufacturer’s instructions (TEL-TEST B, Inc., Friendswood, TX). Total cDNA was amplified with random hexamers (Invitrogen) and then detected using standard PCR. Forward primer Lpg2647 RT-F (5′-GCGCACGATCCATGAATTTACCCA-3′) and reverse primer Lpg2647 RT-R (5′-TCGGATTGACCAGCTACAACCTGT-3′) were used to assess *phhA* transcription, revealing the presence of a 101-bp product. In order to have a positive control, transcription of 16S rRNA was monitored using primers OR113 (5′- AAAGGGTGCGTAGGTGGTTGATTA-3′ and OR114 (5′-GGTTGCGCTCGTTACGGGACTTA-3′). Primers LetA-F (5′-ATACGACATCAGGGGAGTGG-3′) and LetA-R (5′-TAGAATGGGCATTCGACGAT-3′) were used to examine *letA* transcription. To assess potential co-transcription of *phhA* and *letA*, primers phhA-letA-F (5′-ATACGACATCAGGGGAGTGG-3′) and phhA-letA-R (5′-TCGGATTGACCAGCTACAACCTGT-3′) were employed. Control experiments in which reverse transcriptase was omitted from the reaction were done to rule out contributions from contaminating DNA in the DNase-treated samples. Relative, end-point PCR reactions were separated by agarose-gel electrophoresis and detected with ethidium bromide staining.

### Preparation of Lysates of *L. pneumophila* Cultures Grown with and without Fe Supplementation

*L. pneumophila* 130b was grown in triplicate 30 ml cultures at 37°C until they reached stationary phase in BYE (∼18 hours) with and without added iron. The concentration of Fe in BYE without Fe supplementation is approximately 13 µM (BYÊlow Fe), and 1.3 mM with the standard Fe supplement (BYÊhigh Fe). After centrifugation of the culture, the supernatant was used for pigment assessment while the pellet was resuspended in 0.75 ml ice-cold sucrose buffer (50 mM Tris-HCl, pH 8, 1 M sucrose, containing complete EDTA-free protease inhibitor cocktail tablets (1 tablet/10 ml) from Roche (Roche Molecular Diagnostics, Mannheim, Germany). To make spheroplasts, the resuspended culture was incubated with EDTA (1 mM) and lysozyme for 30 minutes followed by addition of 20 mM MgSO_4_. The spheroplasts were pelleted by centrifugation (5000×g, 20 minutes) and resuspended in 2.5 ml cold 50 mM Tris-HCl, pH 8, with protease inhibitors (Roche) before being lysed by three rounds of 15-s sonication (15% output on a model 450 Branson sonifier). Unlysed bacteria and cellular debris were removed by a 10-min centrifugation at 5000×g. The bacterial lysate was frozen in a dry ice/ethanol bath and stored at −80°C. In order to measure PAH activity in the bacterial lysates, these were thawed and run through a Zeba Spin Desalting Column (Thermo Scientific) to remove free amino acids and other small molecular weight compounds, followed by determination of total protein concentration by the Bio-Rad Protein Assay.

### Quantitative RT-PCR Analysis of *L. pneumophila* Gene Expression in BYE with and without Fe Supplementation

cDNA templates were obtained from 1 µg total RNA using random primers (Invitrogen) and SuperScript III reverse transcriptase (Invitrogen) according to manufacturer’s instructions. Quantitative RT-PCR was performed on a My IQ2 (Bio-Rad Laboratories, Hercules, CA, USA) cycler using IQ SYBR Green Supermix (Bio-Rad) according to the manufacturer’s instructions. The primers used to assess *phhA* and *letA* were as noted above. Primers used for assessing *lbtA* were as described before (see [35]). The *gyrB* gene was used as a reference gene to normalize gene expression. The primers used for *gyrB* assessment were 5′-AATCCCACTGCAGCAAAATC-3′ and 5′-TGGTAAACCGGCAATATCCA-3′. The level of gene expression was assessed by determining the cycle at which the amplification curve crossed the detection threshold. The relative expression was calculated using the DCT method, where DCT = CT gene – CT reference gene (both *16S* and *gyrB*), where CT is cycle threshold. The relative change in gene expression was calculated using the 2DDCT, where DDCT = DCT BYÊlow Fe sample – DCT BYÊhigh Fe sample.

### Cloning of *phhA* from *L. pneumophila* for Isolation of lpPAH

The gene encoding PAH in *L. pneumophila* Philadelphia 1 (lpg2647 or *phhA*) was amplified from genomic DNA using the following primers based on the GenBank sequence (gi: 52842853): 5′-GGGAATTCCATATGAATGAGATGAGTGAAGGAGAG-3′ and 5′-CCTTAAGCTCGAGACAAGCCCTTATATGAATATTTG-3′. The *Nde*I and *Xho*I restriction sites are underlined. The resulting 849 kb fragment was cloned into pGEM-T Easy Vector (Promega). After cloning it was discovered that this sequence encodes a protein with 9 extra amino acids in the N-terminal (MNEMSEGEI). Based on comparison with *phhA* from other *L. pneumophila* strains (lpc0492, lpl2572 and lpp2700) and determination of a putative ribosome binding-site using GeneMark™ [37], [38], we reported the shorter sequence to NCBI, which subsequently annotated it in GenBank (gi: 224458591) (Fig. S1). To obtain the correct amino acid sequence upon expression, the pGEM/lpg2647 vector was mutated to contain a second *Nde*I cutting site just 5′ to the correct start codon using the following mutagenic primer: 5′-GATGAGTGAAGGAGAGCATATGGAGTTTAGTAGCC-3′. The mutation was verified by sequencing before the plasmids were digested with *Nde*I and *Xho*I to excise *phhA*. The 818 kb DNA fragment was gel purified and ligated into pET-30a (pET-30a/lpg2647) to enable expression of *L. pneumophila* PAH (lpPAH) with a C-terminal-tag (lpPAH-(HIS)_6_). To enable expression with a cleavable N-terminal tag, pET30-a/lpg2647 was used as template for amplification of *phhA* by primers 5′-GCTTCCATGGAGTTTAGTAGCCGGTAT-3′) and 5′-GCTTGGTACCTTACAGTCAGTCA-3′. The underlined restriction sites (*Acc65I* and *NcoI*, respectively) were used to transfer the gene to the pET-ZZ-1a vector [39], coding for a fusion protein (HIS)_6_-ZZ-lpPAH, with a Tev-cutting site between the ZZ carrier and lpPAH. The primers used for mutagenesis were provided by MWG Biotech AG (Ebersberg, Germany) and DNA sequencing was done at the Sequencing Facility of the University of Bergen.

### Overexpression and Purification of lpPAH

Both variants of recombinant PAH from *L. pneumophila* (lpPAH), i.e. lpPAH(HIS)_6_ and (HIS)_6_-ZZ-lpPAH, were produced by *E. coli* strain BL21-Codon Plus(DE3)RIL in LB with IPTG-induction (1 mM) or autoinduction medium as previously described [40]. All media were supplemented with kanamycin (LB:30 µg/ml, autoinduction medium: 100 µg/ml). The cells were disrupted using sonication or French press and the soluble fraction of the crude extract was applied to TALON® resin (Clontech) equilibrated with wash buffer (50 mM Na-phosphate, 300 mM NaCl, pH 7.0), at 4°C. Removal of unbound proteins was monitored by measuring the absorbance at 280 nm (about 10 column volumes) and the lpPAH-(HIS)_6_ or the (HIS)_6_-ZZ-lpPAH fusion protein was eluted with 3 column volumes of elution buffer (50 mM Na-phosphate, 200 mM NaCl, pH 7.0, 150 mM imidazole). Buffer-exchange to 20 mM Na-Hepes, 200 mM NaCl, pH 7, was either done by equilibrated PD-10 columns or by size-exclusion chromatography on a HiLoad Superdex 200 column (GE Healthcare). The (HIS)_6_-ZZ-lpPAH fusion protein was cut with (His)_6_-tagged TEV overnight at 4°C and the isolated lpPAH was obtained by collecting the flow-through from a second TALON column. Size exclusion chromatography on a calibrated HiLoad Superdex 200 (1.6 cm×60 cm) column was also used to estimate the molecular size of lpPAH-(His)_6_ and lpPAH using a flow of 1 ml/min. Protein concentration of cpPAH was measured spectrophotometrically using the extinction coefficient ε_280_ = 1.20 (mg/mL)^−1^ cm^−1^, calculated using the method of Pace et al. [41]. Iron content in isolated lpPAH was determined by chelation with bathophenantrolinedisulfonic acid (BPDA), by measuring the formation of the BPDA–Fe(II) complex spectrophotometrically (ε_535_ = 22 000 M^−1^ cm^−1^), essentially as described in [42]. The purified protein was stored in liquid nitrogen.

### PAH Activity Measurements

PAH activity was customarily measured for 1 min at 37°C using 0.25 µg of purified lpPAH-(His)_6_. In standard assays, the enzyme was incubated with 1 mM L-Phe in 100 mM Na-Hepes, pH 7, 0.04 mg/ml catalase and 0.5% bovine serum albumin. After 4 minutes incubation, 100 µM ferrous ammonium sulphate (FAS) was added and further incubated for 1 min before the reaction was started by adding 0.2 mM BH_4_ (from Dr. B. Schircks Laboratories) and 5 mM DTT. The reaction was stopped after 1 min by adding 1% (by vol) acetic acid in ethanol (1∶1) and subsequent freezing, and precipitated protein was removed by centrifugation (12,000×*g*, for 5 min). The amount of tyrosine produced was measured after separation by HPLC and fluorimetric detection [43], [44]. *V*_max_ and *K*_m_ values for the cofactor BH_4_, the substrate L-Phe and iron were obtained by activity measurements with various concentrations of cofactor up to 500 µM (with 1 mM L-Phe and 100 µM FAS) or with various concentrations of L-Phe up to 5 mM (with 200 µM BH_4_ and 100 µM FAS), or with various concentrations of FAS up to 500 µM (with 1 mM L-Phe and 200 µM BH_4_). The saturation curves were fitted to a hyperbolic Michaelis–Menten equation using Sigma Plot v.9.0. (SPSS Inc). The effect of temperature on PAH activity was measured by preincubating the enzyme (5 min) in 100 mM Na-Hepes, pH 7, 0.04 mg/ml catalase and 0.5% bovine serum albumin at increasing temperatures from 6 to 65°C before measuring the activity at the same temperature. To assess the thermal stability of the enzyme, lpPAH was incubated for 10 min at different temperatures in the range 0 to 70°C. Immediately after the thermal incubation, the enzyme was placed at 37°C and left to equilibrate at the lowered temperature during assay preincubation (5 min) before the activity was measured at standard conditions.

Prior to performing PAH activity measurements in crude extract of *L. pneumophila* 130b, the frozen lysates were thawed and run through Zeba mini centrifugal buffer exchange columns (Pierce) to remove low molecular weight compounds. 20 µl of the lysate was assayed at otherwise similar conditions as for the recombinant lpPAH. When indicated, the assay was also performed in the absence of added iron.

### Dynamic Light Scattering (DLS)

Hydrodynamic radius and polydispersity were estimated by DLS using a Nanosizer S (Malvern Instruments, Sweden) and a 12 µl-quartz cuvette (Malvern) with protein samples (1 mg/ml) prepared in 20 mM Na-Hepes, 200 mM NaCl, pH 7.0 and centrifuged (10 600×g for 10 min, 4°C).

### Characterization of the Conformation and Thermal Stability by Circular Dichroism (CD) and Differential Scanning Calorimetry (DSC)

CD was performed on a Jasco J-810 spectropolarimeter equipped with a Jasco 423S Peltier element for temperature control, with 6 µM lpPAH subunit in freshly made and degassed buffer. For wavelength scans, the protein was measured in 50 mM Na-phosphate buffer, pH 6.5. Stoichiometric amounts of FAS were added prior to measurements unless otherwise indicated. The samples were degassed for 5–10 min at 37°C prior to acquisition of the CD spectra at the same temperature. CD-monitored thermal denaturation was performed by following the changes in ellipticity at 222 nm, with a scan rate of 1 K/min in the range 37–100°C in the indicated buffer. The apparent melting temperature (*T*_m_) of lpPAH was also determined by DSC using a MicroCal VP-DSC microcalorimeter (GE Healthcare). A degassed sample of 30 µM lpPAH in 20 mM Na-Hepes, pH 7.0 with or without 200 mM NaCl, as indicated, was heated at a scan rate of 1 K/min. The enzyme was preincubated with a stoichiometric amount of freshly prepared iron (as FAS). A buffer-buffer scan was subtracted and a rescan was performed to determine reversibility.

## Results

### Identification and Transcription of the *L. pneumophila phhA* Gene

**Figure 2:**
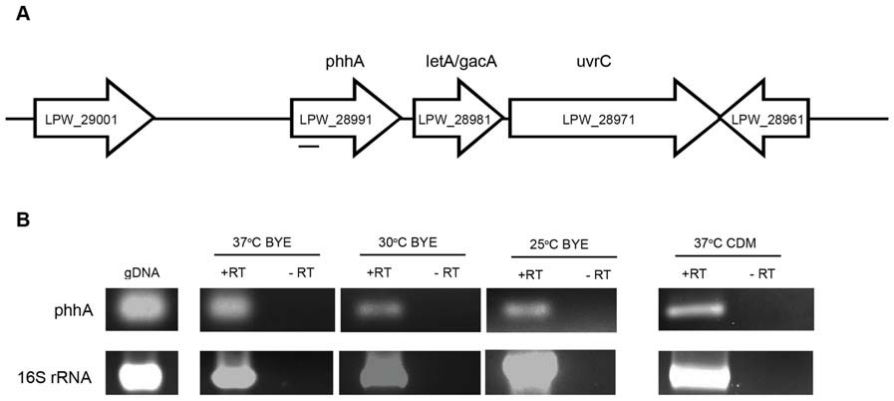
Location and expression of *phhA*. (A) Depiction of the region of the *L. pneumophila* chromosome containing *phhA*. The white horizontal arrows denote the relative size and orientation of *phhA* and its neighboring genes. The thin horizontal line below the gene map signifies the approximate size and location of the *phhA*-specific transcript identified by RT-PCR analysis. (B) Expression of *phhA* transcripts. Wild-type strain 130b was grown in BYE or CDM broth at the indicated temperatures, and then RNA was analyzed by RT-PCR utilizing primers specific to *phhA*. That the PCR products obtained resulted from mRNA templates was confirmed by the lack of product obtained when the PCR did not incorporate RT. PCR products obtained from genomic DNA appear in the left-most lane, indicating that the mRNAs observed are full-length. RT-PCR analysis of 16S rRNA served as a positive control. The results presented are representative of at least three independent experiments.

**Figure 3:**
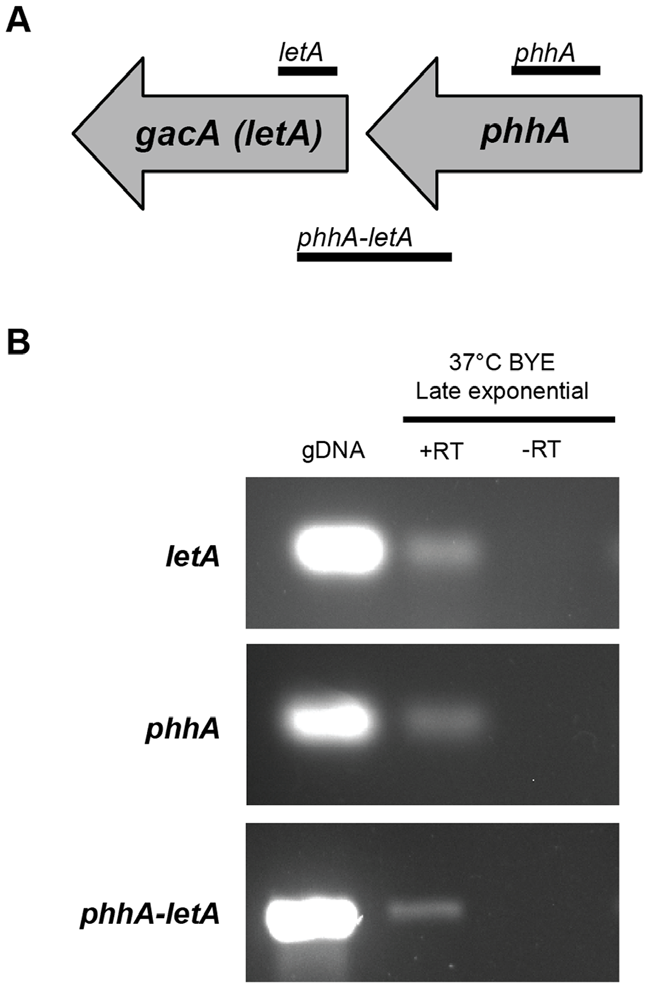
Transcriptional linkage between *phhA* and *letA*. (A) The gray horizontal arrows denote the *phhA* and *letA* genes. The thin horizontal lines below the genes signify the approximate size and location of transcripts identified by RT-PCR analysis, including an intergenic transcript. (B) Wild-type strain 130b was grown in BYE at 37°C, and then RNA was analyzed by RT-PCR utilizing primer pairs specific to either *phhA, letA,* or the intergenic region spanning *phhA* and *letA*. That the PCR products obtained resulted from mRNA templates was confirmed by the lack of product obtained when the PCR did not incorporate RT (-RT). PCR products obtained from genomic DNA appear in the left-most lanes, indicating that the mRNAs observed are full-length. The results presented are representative of at least three independent experiments.

**Figure 4:**
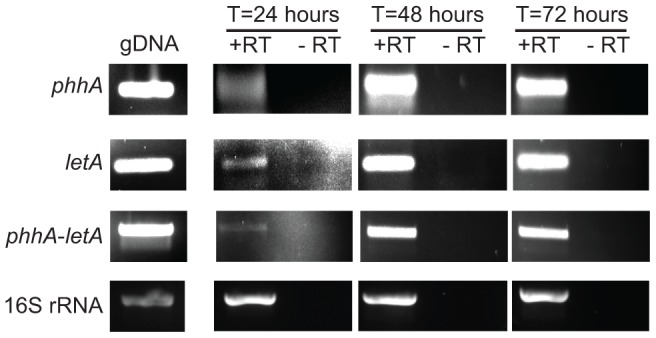
Intracellular expression of *phhA* and growth of *L. pneumophila* in U937 cell macrophages. Intracellular expression of *phhA* and *letA* transcripts in macrophages, which were infected with wild-type (WT) 130b for 24 h, 48 and 72 h and then RT-PCR was done using primers that amplify the specific transcripts. That the PCR products obtained resulted from mRNA templates was confirmed by the lack of product obtained when the PCR did not incorporate reverse transcriptase (- RT). The results are representative of two independent experiments.

Examination of the *L. pneumophila* genome databases revealed that the organism contains a gene (*phhA*) that is predicted to encode a PAH. In the genome sequence of strain 130b [45] the *phhA* gene is designated as ORF lpw_28991 and maps as the first gene in a putative 3-gene operon with the two downstream genes being *letA* (*Legionella* transmission activator, also known as *gacA*) and *uvrC* (excinuclease UvrABC subunit C) (Fig. 2A). In the other *L. pneumophila* databases [46], [47], [48], *phhA* is denoted as lpp2700, lpg2647, lpl2572, lpc_0492, and lpa_03875 for strains Paris, Philadelphia, Lens, Corby, and Alcoy, respectively, and in all cases, the gene has the same nearest neighbors. The *phhA* gene was predicted to encode a 272-aa protein (see Fig. S1) that is 98–100% identical between the six sequenced strains of *L. pneumophila* and 38–46% identical to the other bacterial PAHs that have been structurally characterized. RT-PCR analysis indicated that the *phhA* gene is expressed by strain 130b when growing in BYE broth, the yeast extract medium standardly used to grow *L. pneumophila* in the laboratory (Fig. 2B). The gene was expressed in late-exponential BYE cultures incubated at 37°C, 30°C, or 25°C. Transcription of *phhA* was also evident, when strain 130b was grown in CDM, a chemically-defined medium that consists of the L-amino acids, nine trace metals, iron, pyruvate, glutathione, α-ketoglutarate, salt, and buffers (Fig. 2B). Moreover, RT-PCR analysis also confirmed that *phhA* is transcriptionally linked to *letA* (Fig. 3). Finally, further RT-PCR analysis detected *phhA* mRNA in *L. pneumophila* growing in U937 macrophages, with expression at 24, 48 and 72 h post infection, and the expression of *letA* was also detected (Figure 4). The *phhA* gene is thus highly conserved within the *L. pneumophila* species and is expressed by *L. pneumophila* grown in a variety of conditions.

### PAH is Necessary for *L. pneumophila* Growth in Media Lacking Tyrosine

**Figure 5:**
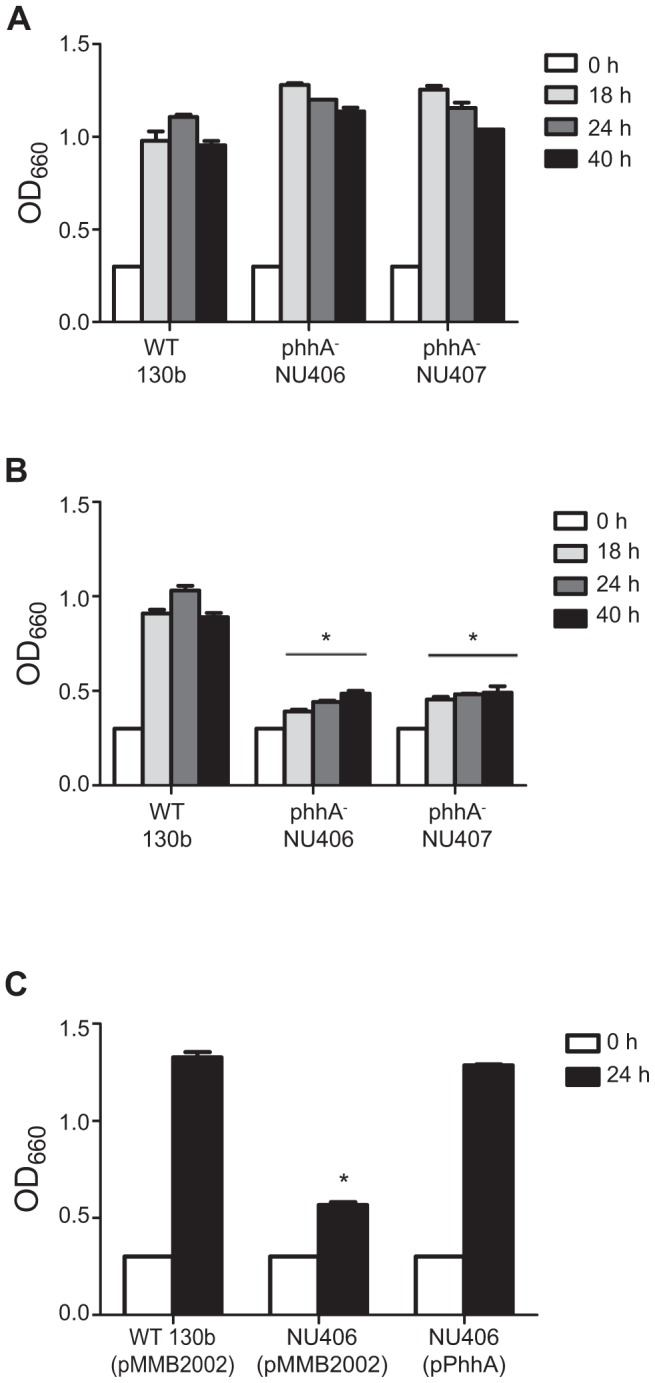
Growth of wild-type and *phhA* mutant *L. pneumophila* in CDM containing different amounts of tyrosine. *L. pneumophila* 130b wild-type (WT) and *phhA* mutant strains NU406 and NU407 were inoculated into either standard CDM containing tyrosine (A) or CDM lacking tyrosine (B) and at the indicated time points, the extent of bacterial growth was determined by recording the optical density (OD) of the cultures. In panel (B), the ODs of the mutant cultures were significantly less than that of the WT cultures at 18, 24, and 40 h post-inoculation, as indicated by the asterisks (*P*<0.05, Student’s *t*-test). (C) WT and mutant NU406 carrying the empty vector (pMMB2002) and NU406 carrying the *phhA* gene cloned into pMMB2002 (i.e., pPhhA) were cultured in CDM without the tyrosine supplement and then at 24 h post-inoculation the OD of the cultures were determined. The OD of the NU406 (pMMB2002) culture was significantly less than that of both the WT and the complemented mutant (*P*<0.05, Student’s *t*-test). All experiments are representative of three independent trials.

In order to investigate the role of PAH in the growth and physiology of *L. pneumophila*, we isolated and characterized two independently derived mutants (NU406 and NU407) of strain 130b that are specifically lacking an intact *phhA* gene. Both of these mutants grew normally in BYE broth (data not shown). They also grew, as WT did, when cultured in CDM (Fig. 5A). These data indicate that PAH is not required for *L. pneumophila* growth in rich media or in media containing a full complement of amino acids. However, when the *phhA* mutants were grown in CDM that lacked its tyrosine component, they exhibited markedly reduced growth (Fig. 5B). Full growth was restored, when an intact copy of *phhA* was introduced into the mutants on a plasmid (Fig. 5C). Taken together, these data confirm that PAH has an essential function for the growth of *L. pneumophila* in tyrosine depleted media.

### PAH Promotes Pyomelanin Production by *L. pneumophila*

**Figure 6:**
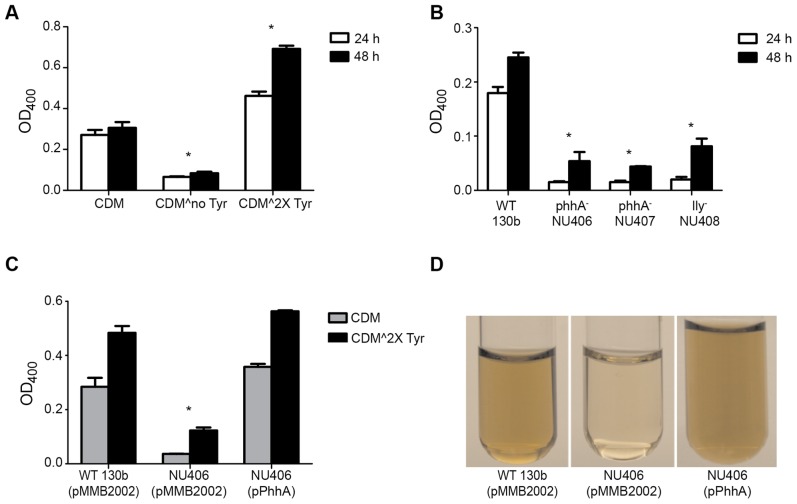
Pigmentation of wild-type and *phhA* mutant *L*. ***pneumophila***. (A) Wild-type (WT) *L. pneumophila* 130b was inoculated into either standard CDM, CDM lacking tyrosine (CDMˆno Tyr), or CDM containing twice the normal amount of tyrosine (CDMˆ2X Tyr), and then at 24 and 48 h post-inoculation the levels of pigment in the cultures were determined by measuring the OD_400_ of the cultures supernatants. (B) *L. pneumophila* 130b WT, *phhA* mutants NU406 and NU407, and *lly* mutant NU408 were inoculated into standard CDM containing tyrosine, and then at 24 and 48 h, the amount of pigment produced was determined by assessing the OD_400_ of culture supernatants. (C) WT and mutant NU406 carrying the vector pMMB2002 and NU406 carrying the *phhA* gene cloned into pMMB2002 (i.e., pPhhA) were cultured in either standard CDM containing tyrosine (gray bars) or CDM containing twice the normal amount of tyrosine (black bars), and then at 24 h post-inoculation the OD_400_ of the cultures were determined. (D) Photographs of cell-free supernatants from 24-h CDM cultures (with the standard amount of tyrosine) of WT and *phhA* mutant strain NU406 carrying vector and NU406 carrying the cloned *phhA*. In panels (A – C), the asterisks indicate a *P* value of <0.05 compared to the WT control (Student’s *t*-test). All experiments are representative of three independent trials.

*L. pneumophila* secretes a brown pigment when it is grown in bacteriological media [19]. This pigment results from the oxidative polymerization of HGA, which is secreted into the culture supernatant [49]. HGA, in turn, is produced via the action of 4-hydroxyphenylpyruvate dioxygenase/legiolysin (HPPD/Lly) [49] (Fig. 1). Thus, the pigment of *L. pneumophila* is best described as a pyomelanin or HGA-melanin. It has been long known that the production of the *L. pneumophila* pigment is tyrosine-dependent [50], and this was confirmed for strain 130b, when we cultured the strain in CDM containing either the standard amount of tyrosine, no tyrosine, or twice the normal amount of tyrosine (Fig. 6A). Therefore, we hypothesized that PAH would help promote pyomelanin production by *L. pneumophila*, by providing an additional source of tyrosine through hydroxylation of phenylalanine in the medium. To address this hypothesis, we grew WT 130b and the *phhA* mutants in standard CDM and then compared the culture supernatants for their absorbance at 400 nm [19]. As predicted, the *phhA* mutants exhibited a dramatic reduction in pigmentation, and in fact, they were as defective as the *lly* mutant was in this growth condition (Fig. 6B). This defect was entirely absent from a complemented *phhA* mutant (Fig. 6C), confirming the important role that lpPAH has in pigment production. These changes in pigmentation could also be observed by eye (Fig. 6D). When the bacteria were grown in CDM containing twice the normal amount of tyrosine, we observed some pigmentation in the *phhA* mutant cultures (Fig. 6C), indicating that PAH, although a facilitator of pyomelanin production, is not absolutely required if sufficient levels of pre-formed tyrosine are present.

### PAH Activity in Bacterial Lysates

**Figure 7:**
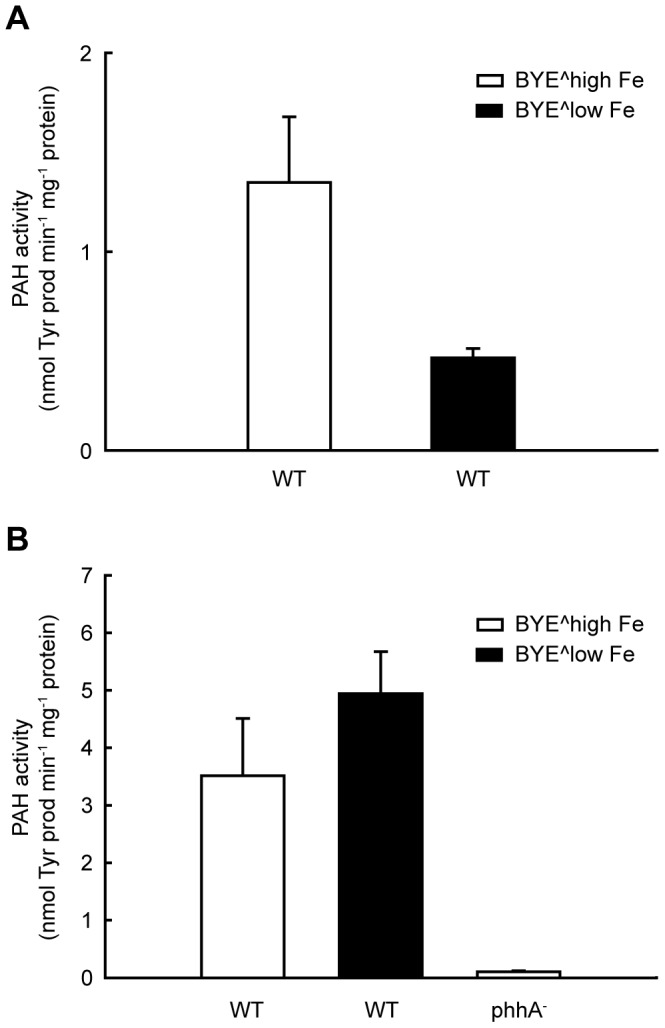
PAH activity in lysates of *L. pneumophila* 130b. PAH activity measured without (A) and with (B) the addition of 100 µM Fe(II) in the assay in lysates from wild-type (WT) cultures grown in BYE medium with (BYÊhigh Fe; white bars) and without (BYÊlow Fe; black bars) standard (1.3 mM) FeCl_3_ supplement (see text for details). The data presented are the means and SD from triplicate measurements of duplicate cultures for each condition. The activity of a lysate of a culture of *phhA* mutant grown in BYÊhigh Fe is also shown in (B). Differences between conditions were found to be significant (P<0.01) with respect to WT in BYÊhigh Fe, in both (A) and (B).

Due to the large background of amino acids, metals and other small compounds in bacterial lysates, filtration is a necessary step before measurement of PAH activity is feasible. After filtration, the activity of the lysates could be measured at standard concentration of reactants (1 mM L-Phe and 0.2 mM BH_4_). When growing the bacteria in BYE without supplemented iron (BYÊlow Fe), very little pigment is produced (OD_400nm_ = 0.023±0.006), while when supplementing the culture media with the standard 1.3 mM FeCl_3_ (BYÊhigh Fe), the pigment production is substantially increased (OD_400nm_ = 0.142±0.027). We also found a similar relation between the specific PAH activity in lysates from low-iron (BYÊlow Fe) and high-iron cultures (BYÊhigh Fe) when the activity was measured in the absence of iron addition to the assay (Fig. 7A). But PAH is dependent on Fe(II) for activity and the addition of 100 µM Fe(II) in the assay resulted in increased PAH activity (Fig. 7B), suggesting that the active site of lpPAH is subsaturated with catalytic iron intracellularly. Moreover, in the presence of Fe(II) in the assays we measured a two-fold higher PAH activity in lysates from the cultures in BYÊlow Fe than in the BYÊhigh Fe (Fig. 7B). As seen by quantitative RT-PCR analysis, however, the higher specific activity of the former lysates did not seem to be caused by a higher amount of total lpPAH protein associated to upregulation of *phhA* expression at low iron concentrations (Fig. S2).

### Expression, Purification and Molecular Characterization of Recombinant lpPAH

After establishing the important role of lpPAH in the synthesis of pyomelanin and in the growth of *L. pneumophila* in media deficient in tyrosine, we aimed to characterize the molecular and kinetic properties of the enzyme. The product of the *phhA* gene was cloned both in pET-30a and pET-ZZ-1a vectors. The corresponding recombinant lpPAH-(His)_6_ and lpPAH were successfully expressed and purified with typical yields of 50 mg/L and 20 mg/L, respectively. As the His-tag was not found to affect the molecular or kinetic properties of the enzyme, lpPAH-(His)_6_ was used for most of the analyses. The expression and purification was monitored by SDS-PAGE (Fig. S3A), also showing that recombinant lpPAH migrates close to 31 kDa, correlating well with its theoretical size (32.5 kDa for the lpPAH-(His)_6_). As determined by BPDA quantification of the iron bound, lpPAH was isolated largely as an apoenzyme (0.07±0.03 mol Fe(II)/mol subunit).

When lpPAH was loaded on a calibrated size exclusion chromatography column (HiLoad Superdex 200), negligible protein eluted in the void volume, indicating the absence of aggregation. Remarkably, the elution volume of the protein corresponded to an apparent molecular weight of 64 kDa (Fig. S3B), indicative of a dimeric structure. When analyzed by the same size exclusion chromatography system and conditions, both the truncated form of human PAH including the catalytic domain (i.e. dimeric hPAH(Glyl03-Gln428)) and monomeric cpPAH, eluted at their corresponding position for 70.0 kDa and 30.7 kDa, respectively [23], [51]. lpPAH was further analyzed by DLS, that provided an estimated hydrodynamic diameter of 8.1±0.1 nm for the enzyme as isolated, which is not significantly altered by Fe(II) addition. By comparative DLS analysis the estimated diameter of monomeric cpPAH was ∼6.0 nm. All together our results indicate that lpPAH presents a larger apparent size than expected from its subunit molecular weight, and compatible with a dimeric structure. Other bacterial PAHs characterized so far are monomeric [22], [23], [52].

### Thermal Stability of lpPAH; Effect of L-Phe and Iron

**Figure 8:**
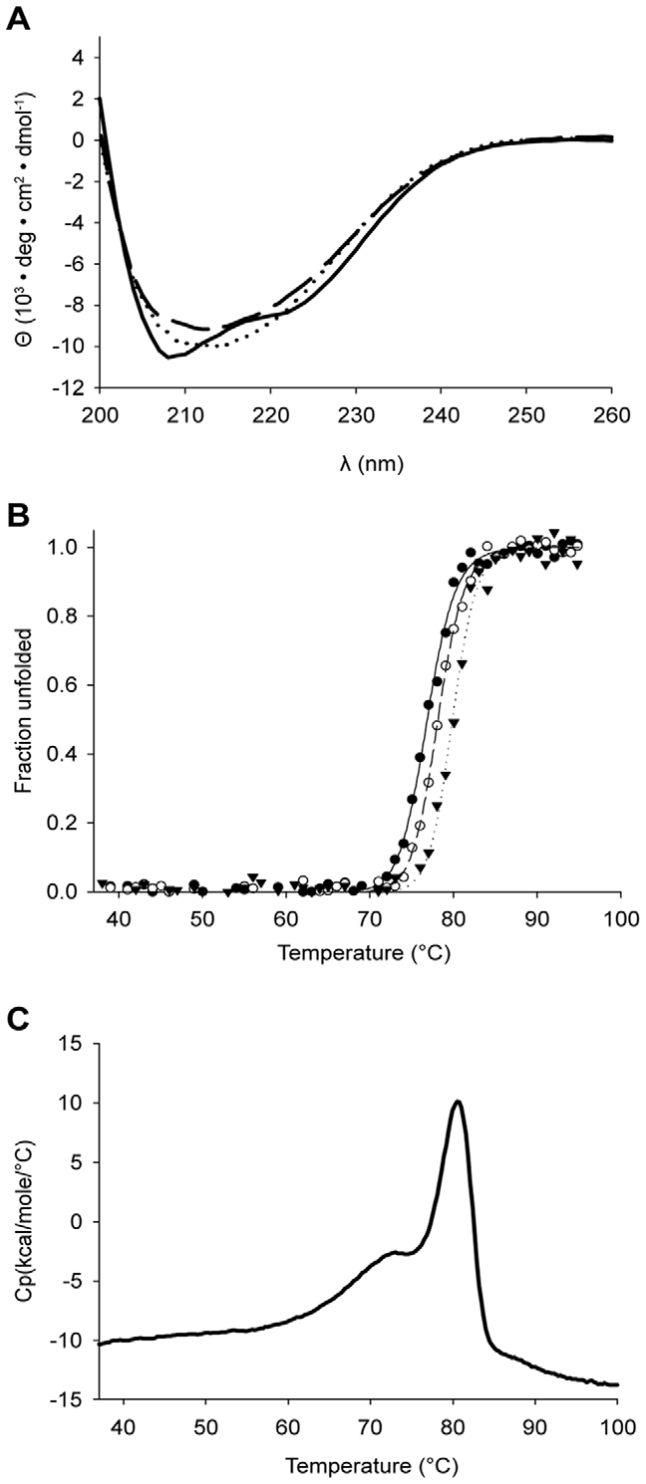
Conformational stability of lpPAH. (A) Far-UV CD spectrum of lpPAH (6 µM in 50 mM Na-phosphate buffer, pH 6.5) at 37°C (**^____^**), at 85°C (––) and at 37°C after heating the sample to 100°C (⋅⋅⋅⋅⋅). [θ], mean residual ellipticity. (B) CD-monitored (at 222 nm) thermal denaturation lpPAH (6 µM in 20 mM Na-Hepes, 200 mM NaCl, pH 7.0) without (•) or with (○) 6 µM Fe(II) (added as ferrous ammonium sulphate) and 6 µM Fe(II) and 5 mM L-Phe (▾). The lines show a fitting of the data to a two-state unfolding equation [79] and points are averaged over ten data points after conversion to fraction unfolded [80]. (C) DSC-monitored thermal denaturation of lpPAH (30 µM) in 20 mM Na-Hepes, pH 7.0. The scan rate was 1°C/min.

The far-UV CD spectrum of lpPAH, recorded at 37°C, shows two minima at 208 and 222 nm, characteristic of proteins with high α-helical content, and similar to other mammalian and bacterial PAHs [23], [53] (Fig. 8A). Processing the spectrum with the program CDNN [54] provided a 33.7 (±1.4)% α-helix and 34.4 (±1.9)% β-strand content. The CD spectrum of lpPAH recorded at 85°C in 50 mM Na-phosphate buffer, pH 6.5 provided a high degree of remaining secondary structure (Fig. 8A), corresponding to about 26.6 (±1.1)% α-helix and 46.9 (±2.6)% β-strand. Moreover, after heating the protein at 100°C the CD spectrum at 37°C also showed that the protein still was largely structured (Fig. 8A). For samples of lpPAH in 20 mM Na-Hepes, 200 mM NaCl, pH 7.0, the CD spectrum obtained at 37°C is similar to that shown in Fig. 8A, but the signal-to-noise ratio is lower (data not shown). Moreover, in the presence of NaCl, the protein irreversibly aggregated at high temperatures and provided CD-monitored denaturation transitions (measured at 222 nm) with high midpoint temperature, *T_m_* (76.9±0.1°C) (Fig. 8B). The effect of ligands was then investigated; addition of stoichiometric amounts of Fe(II) did not affect the CD spectrum or the estimated α-helical content of lpPAH but the *T_m_* values obtained by thermal dependent CD (in the presence of 200 mM NaCl) increased by ∼1.4°C (Fig. 8B). The combined addition of stoichiometric Fe(II) (6 µM) and substrate L-Phe (5 mM) up-shifted the *T_m_* by ∼3°C (Fig. 8B), while addition of superstoichiometric (17-fold) Fe(II) only increased the *T*_m_ by ∼1.9°C (data not shown).

The high midpoint denaturation temperature of lpPAH, indicative of a thermostable enzyme, was then corroborated by DSC, which for the enzyme at pH 7.0 in the absence of NaCl provided an endothermic transition with calorimetric enthalpy change (Δ*H*) = 169.9±0.2 kcal/mol and *T*_m_ = 79±0.5°C, with a shoulder at lower *T*_m_ (Fig. 8C). In the presence of NaCl, the posttransition baseline is kinetically distorted due to aggregation of the sample and does not allow estimations of the *T*_m_-value (data not shown).

### Kinetic Characterization of lpPAH

**Figure 9:**
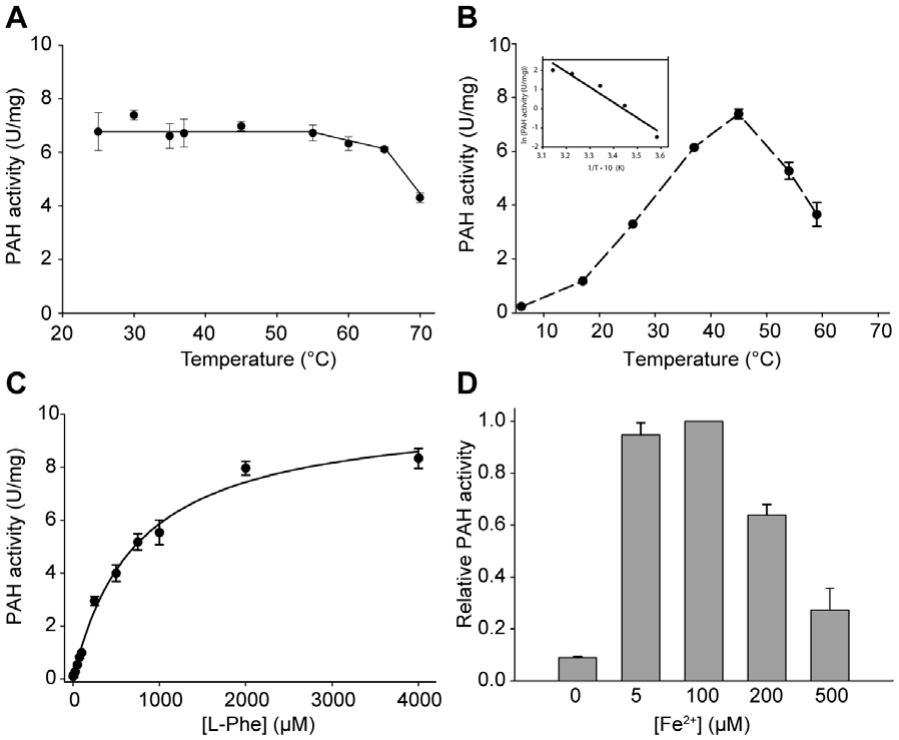
Steady-state kinetic characterization of lpPAH. (A) Residual activity at 37°C after incubation for 10 min at the indicated temperatures. (B) Temperature dependence of lpPAH activity. Inset, Arrhenius plot (providing an activation energy (*E*_a_) of 15.8±2.3 kcal/mol). (C) Effect of L-Phe concentration on lpPAH activity, measured with 0.2 mM BH_4_ and 100 µM Fe(II). The solid line is a fitting to the Michaelis-Menten equation. (D) Effect of Fe(II)-concentration on lpPAH activity, measured with 1 mM L-Phe and 0.2 mM BH_4_. One enzyme unit (U) corresponds to the amount of enzyme that catalyzes the production of 1 µmol L-Tyr per minute.

The high thermal stability of lpPAH was surprising and we therefore investigated the effect of temperature on the enzymatic activity. The activity of the enzyme at 37°C was largely maintained after 10 min preincubation up to 60°C (Fig. 9A) and maximal activity at the selected concentrations of substrate and cofactor was obtained around 45°C (Fig. 9B). This temperature is lower than the characteristic maximal temperatures for typical thermophilic enzymes [55], but the activation energy of the catalyzed reaction is relatively high (*E*_a_ = 15.8±2.3 kcal/mol) (Fig. 9B, inset), in fact 3-fold higher than for cold-adapted cpPAH (*E*_a_ = 4.9±0.3 kcal/mol), in agreement with the relation between *E*_a_ and temperature adaptation [56].

Mammalian PAH is activated by preincubation with L-Phe, which is associated to the positive regulation and cooperativity exerted by the substrate [9], [57], while we have previously reported that the activity of cpPAH is reduced eight-fold when preincubated with L-Phe, which binds with apparent positive cooperativity [23]. This inactivation in cpPAH is most probably caused by long-lived dead-end complexes that temporarily prevent the binding of cofactor BH_4_ before the sequential binding of L-Phe [23]. LpPAH, however, is not affected in any direction by preincubation with the substrate, and the activity exhibited hyperbolic dependence on L-Phe concentration (Fig. 9C). Moreover, lpPAH also showed low affinity for its substrate and cofactor at 37°C (Table 1), with larger *K*_m_-values than those obtained with PAH from bacteria [58], *Caenorhabditis elegans* [59], *Dictyostelium discoideum* [60] and human [44], for which *K*_m_ range from 60 to 620 µM for L-Phe and 30 to 50 µM for BH_4_. Finally, almost no activity was measured in the absence of added iron in the assay (Fig. 9D), further corroborating that the enzyme was isolated largely as an apoenzyme, while inhibition by iron was observed at concentration of Fe(II) >100 µM.

**Table 1:** Steady-state enzyme kinetics parameters for the activity of lpPAH at the selected conditions.

*V*_max_ a	*K*_m_(L-Phe)^a^	*K*_m_(BH_4_)^b^	*C*_0.5_(Fe(II))^c^
µmol L-Tyr ⋅ min^−1^ ⋅ mg^−1^	µM	µM	µM
10.2±0.3	735±50	125±25	1.3±0.5

a Measured at 200 µM BH_4_ in L-Phe saturation curves.

b Measured at 1 mM L-Phe in BH_4_ saturation curves.

c Concentration of Fe(II) that provides half-maximal activity, measured in the range 0–50 µM Fe(II).

## Discussion

### LpPAH and Pyomelanin Synthesis

The important role of pyomelanin as a protective pigment against reactive oxygen intermediates and other environmental stresses and as an agent for iron reduction and uptake is well established [19], [61]. It is believed that these functions provide an adaptive advantage to the pyomelanin-producing pathogenic bacteria, notably in chronic lung infections [15], [62], [63]. It has also long been known that pyomelanin production is stimulated by increased L-Phe and L-Tyr supplementation in several bacteria, including *L. pneumophila* [50]. However, a role of *phhA*/PAH in pyomelanin synthesis has so far not been clear and previous studies have focused on the function of other enzymes in the pigment-synthesis pathway, such as HPPD/Lly and HmgA (Fig. 1) [15], [49], [61], [63], [64]. In a recent study, *phhA* was among the 26 genes identified to contribute to the production of HGA in *P. aeruginosa* [65] and the important functional role of *phhA*/PAH in pyomelanin production in *L. pneumophila* is clearly established in this work. In L-Tyr-depleted media, pyomelanin synthesis as well as the growth of *L. pneumophila* are dependent of *phhA* and, remarkably, the *phhA* mutant does not reach normal values of pigment production even upon supplementation with L-Tyr. In this context it is worth noting that in continuous cultures of *L. pneumophila* at low concentration of oxygen, which is one of the substrates of PAH, tyrosine becomes limiting for bacterial growth [66].

It is also noteworthy that the gene organization around the *phhA* locus *in L. pneumophila* is different to that in other pyomelanin producing bacteria, where *phhA* is connected to *phhB* and *phhC*. *phhB* encodes pterin-4a-carbinolamine dehydratase (PCD), an enzyme involved in recycling of tetrahydropterins, while *phhC* encodes an aromatic amino acid transferase (AT) [12]. In the *L. pneumophila* genome there is no homologue to *P. aeruginosa phhC,* and *phhB* is next to *hisC2* which encodes an AT that could perform this intermediate step in the pathway (Fig. 1). The genomic position of the gene next to the dehydratase supports this role of *hisC2*.

In *L. pneumophila*, *phhA* is connected to *letA (gacA)* and *uvrC*, which encode a regulator of the expression of proteins characteristic of the transmission phenotype – and activates virulence - (LetA), and subunit C of an exonuclease protecting from UV-damage (UvrC) [67], [68]. In fact, as shown in this work, *letA* and *phhA* are transcriptionally linked (Fig. 3). Interestingly, while it is common for *letA* (also called *GacA, uvrY, sirA* and *luxR)* to be positioned next to *uvrC*, *Legionella* is, to our knowledge, the only genus where *phhA* is in the same operon as these two genes. Thus, it is plausible that regulation of *phhA* expression might be an adaptive advantage for the pathogenesis of *L. pneumophila.* Elucidating the role *phhA* in infection will be an important goal for future investigations.

### Effect of Iron on Pyomelanin Synthesis

Pyomelanin produced by *L. pneumophila* has an essential intrinsic ferric reductase activity, converting Fe(III) to Fe(II) [19]. On the other hand, excessive reduction of iron due to hyperpigmentation is inhibitory for *L. pneumophila* growth [19]. We thus considered a possible regulatory role of iron in *phhA* expression and in lpPAH activity. We found that the activity of lpPAH shows an absolute requirement for Fe(II), in agreement with PAH being a non-heme iron enzyme where the metal has a catalytic function involved in the activation of di-oxygen in concert with the cofactor [69]. And while iron does not seem to be a transcriptional regulator of lpPAH (Fig. S2), its activity is inhibited by high iron concentrations (Fig. 9D) which might explain the lower specific PAH activity in lysates from cultures of *L. pneumophila* grown with iron supplementation (Fig. 7A). In addition to PAH, the pathway from phenylalanine to HGA includes two more iron-containing enzymes, HPPD/Lly and HmgA (Fig. 1), and it has also been reported that HPPD from *Pseudomonas* sp. strain P.J. 874 is inhibited by Fe(II) concentrations >0.3 mM [70].

### The Thermal Stability of lpPAH

The *T*_m_-value for the denaturation of lpPAH is much higher than for other mammalian or mesophilic bacterial PAHs studied so far, clustering around 55°C [50], [71], [72], and comparable to that of the PAH from the thermophile *Chloroflexus aurantiacus* (caPAH) [24]. Interestingly, *L. pneumophila* shows an optimal temperature around 35–37°C but lives at 4–63°C [3]. In fact, *L. pneumophila* tolerates well high temperatures, but cell multiplication and CO_2_ production decrease markedly at temperatures >44–45°C, while cell multiplication generally stops at around 48.4–50.0°C [73], [74]. It therefore appears that the temperature dependence of the activity of lpPAH (Fig. 9B) corresponds well with the growth-range for *L. pneumophila.* At the same time this enzyme shows an ability to preserve its overall structure and activity at higher temperatures, where the bacterium only survives, and might recover activity when returning to lower temperatures. The conformational stability of lpPAH was even preserved as apoenzyme. In fact the recombinant enzyme is expressed as iron free apoenzyme, and iron binding only stabilizes the protein by <2°C. This is different to what has been found for cvPAH and caPAH for which the apoenzyme shows a lower stability and iron leads to a large stabilization (≤12°C) [24], [53], [72]. In general, the degree of stabilization by ligand binding is lower in thermophilic than in psychrophilic and mesophilic homologues [56].

The structural features that determine protein thermostability are varied and include among other increased internal packing, decreased flexibility, and larger number of ion pairs networks (mainly salt bridges), hydrogen bonds, hydrophobic interactions, disulfide bridges, aromatic clusters and oligomeric interactions (for reviews see [55], [75]). The specific and cumulative contribution of these stabilizing features seems to vary for each thermophilic protein species. Although the analysis of specific residues and interactions defining the thermal stability of lpPAH must await the elucidation of its 3D-structure, we noticed two aromatic residues in its amino acid sequence, i.e. Phe150 and Trp155, of particular interest in the context of thermal stabilization. Trp155 is not conserved in any if the bacterial genomes available at present, while the appearance of Phe at equivalent position to Phe150 is rare (Fig. S1 and data not shown). Thermophilic enzymes frequently present increased number of clustered aromatic residues, which are often mutated to Leu in the mesophilic counterparts [76], as is the case in cvPAH (Fig. S1). Surface-exposed small aromatic clusters, often located close to the active sites, have been found to confer an entropic advantage (and improved free energy) over mesophilic analogues through generation of low-frequency motions [77]. Based on the high sequence identity with the other PAH enzymes of known 3D-structure, Trp155 would be located at the start of the helix leading to the iron-coordinating residue Glu167 and might form a stabilizing aromatic cluster with Phe150 and/or Phe92 (a conserved nearby residue). Furthermore, it is also well established that oligomerization is a strong stabilization mechanism and a large proportion of hyperthermophilic proteins have a higher oligomerization state than their mesophilic counterparts (for a review see [75]). In this context it is interesting that thermostable lpPAH appears to be dimeric, while other purified and characterized bacterial PAHs are monomeric [22], [23], [52].

### Other Properties of lpPAH

At 37°C the specific activity of lpPAH is ≥3-fold higher than that of other PAHs from eukaryote and prokaryote organisms [23], [24], [44], [58], [59]. At the same time, lpPAH shows *K*_m_-values for both substrate and cofactor which also are 3-4-fold higher than for other characterized PAHs, providing relatively similar catalytic efficiencies (*k*_cat_/*K*_m_) at 37°C for the enzyme family. Thus, lpPAH appears to be an efficient enzyme only at high substrate levels. One advantage of a low affinity for substrate and cofactor might be that the synthesis of pyomelanin will only occur at high concentrations of the substrate, which will safeguard a threshold value of L-Phe for protein synthesis. Along evolution, more rigorous mechanisms to regulate PAH activity depending on L-Phe concentration have been adopted, such as the proposed additional regulatory binding of L-Phe observed in PAH from *C. elegans* and positive cooperativity in the mammalian enzymes [78].

### Conclusion

In *L. pneumophila*, lpPAH, the product of the *phhA* gene, appears to have an important functional role in the synthesis of the pigment pyomelanin and in the growth of the bacterium in low-tyrosine medium. Furthermore, *phhA* is transcriptionally linked to *letA,* an activator of virulence in *L. pneumophila,* and is expressed in macrophage-infecting bacteria. The molecular and kinetic properties of lpPAH, and notably its thermal stability, correlate well with the adaptive properties of the pathogen *L. pneumophila*.

## Supporting Information

Figure S1 **Alignment of lpPAH with other PAHs.** Cp, *Colwellia psychrerythraea*; lp, *Legionella pneumophila*; pa, *Pseudomonas aeruginosa;* cv, *Chromobacterium violaceum*; h, *Homo sapiens*. Identical residues are denoted by a red background and similar residues in red text. The catalytic iron-coordinating residues are indicated by triangles and other active site residues by circles. Top and bottom secondary structures are derived from PDB ID 2v27 and 1PAH, respectively.
(TIF)

Figure S2 **Quantitative RT-PCR analysis of** ***phhA*** **expression by wild type strain 130b grown in BYE broth in the presence (white bars) and absence (black bars) of the standard iron supplementation.** (A) The level of gene expression in lysates from cultures grown in BYE medium with (BYÊhigh Fe; white bars) and without (BYÊlow Fe; black bars) standard (1.3 mM) FeCl_3_ supplement, was assessed by determining the cycle at which the amplification curve crossed the detection threshold. The relative expression was calculated using the DCT method, where DCT = CT gene – CT reference gene (*gyrB*). (B) The relative change in gene expression was calculated using the 2DDCT, where DDCT = DCT BYÊlow Fe sample – DCT BYÊhigh Fe (BYE) sample. For comparison, the levels of *letA* and *lbtA* expression were also determined; *lbtA* has been previously shown to be repressed during 130b growth in media containing higher amounts of iron [35]. Values are means and standard deviations from three-independent experiments.
(TIF)

Figure S3 **Expression, purification** and size determination **of recombinant lpPAH.** A) SDS-PAGE analysis of the overexpression and purification of lpPAH-(His)_6_ by affinity chromatography. Lane 1, low molecular weight markers; lane 2, uninduced culture; lane 3, induced culture; lane 4, crude extract (soluble fraction); lane 5, flow-through from Talon column; lane 6, eluted protein fraction from Talon column (purified lpPAH). B) Calibration curve for the determination of protein molecular weight by size exclusion chromatography with a HiLoad Superdex column (1.6 cm×60 cm) at a flow rate of 1 ml/min. The black circles represent the positions for the following proteins used for calibration: ferritin (440 kD), aldolase (158 kD), conalbumin (75 kD), carbonic anhydrase (29 kD) and ribonuclease A (13.7 kD). The position of hPAH(Glyl03-Gln428) (dimer of 70 kDa); cpPAH (monomer of 30.7 kDa) and lpPAH (estimated from this calibration to be a dimer of 64.5 kDa) are also shown. V_e_, elution volume; V_o_, void volume.
(TIF)
