# Supplementary figures and images for: Phenylalanine Hydroxylase from Legionella pneumophila Is a Thermostable Enzyme with a Major Functional Role in Pyomelanin Synthesis
Source: PLoS One. 2012 Sep 26;7(9):e46209. doi: 10.1371/journal.pone.0046209 (PMC3458870; doi:10.1371/journal.pone.0046209)

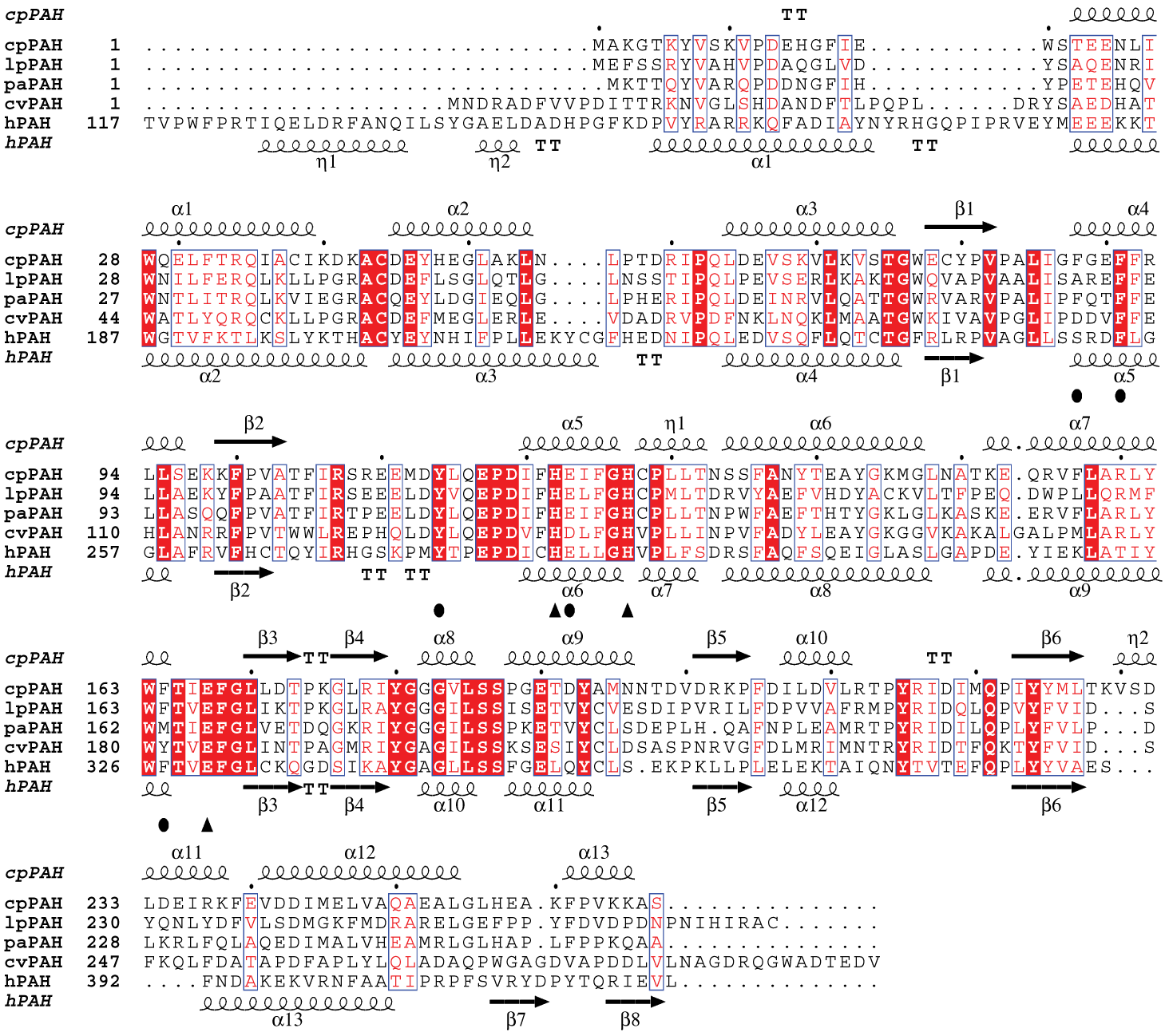

Supplement: Figure S1 — Alignment of lpPAH with other PAHs. Cp, Colwellia psychrerythraea; lp, Legionella pneumophila; pa, Pseudomonas aeruginosa; cv, Chromobacterium violaceum; h, Homo sapiens. Identical residues are denoted by a red background and similar residues in red text. The catalytic iron-coordinating residues are indicated by triangles and other active site residues by circles. Top and bottom secondary structures are derived from PDB ID 2v27 and 1PAH, respectively. (TIF) [file pone.0046209.s001.tif]

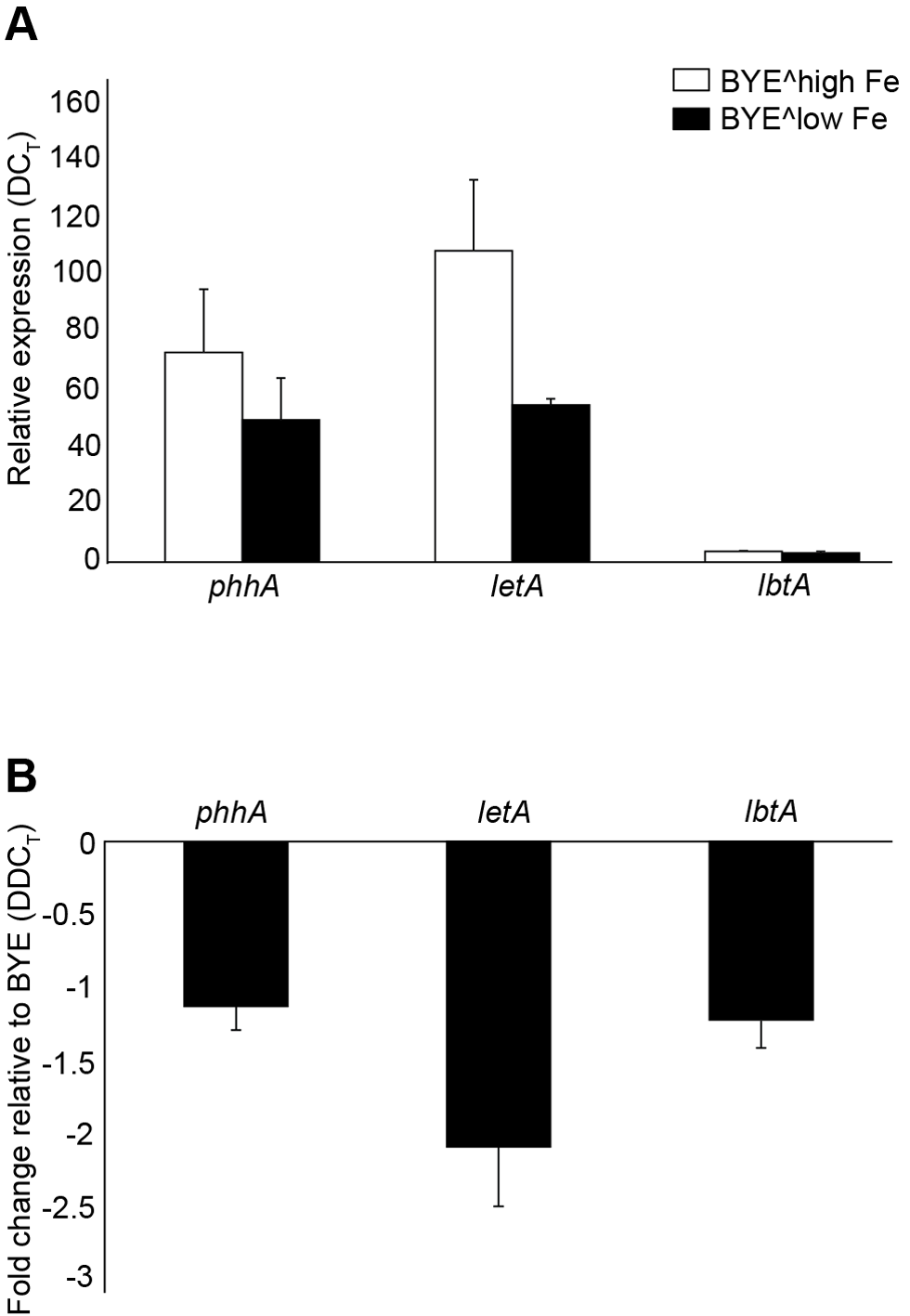

Supplement: Figure S2 — Quantitative RT-PCR analysis of phhA expression by wild type strain 130b grown in BYE broth in the presence (white bars) and absence (black bars) of the standard iron supplementation. (A) The level of gene expression in lysates from cultures grown in BYE medium with (BYÊhigh Fe; white bars) and without (BYÊlow Fe; black bars) standard (1.3 mM) FeCl3 supplement, was assessed by determining the cycle at which the amplification curve crossed the detection threshold. The relative expression was calculated using the DCT method, where DCT = CT gene – CT reference gene (gyrB). (B) The relative change in gene expression was calculated using the 2DDCT, where DDCT = DCT BYÊlow Fe sample – DCT BYÊhigh Fe (BYE) sample. For comparison, the levels of letA and lbtA expression were also determined; lbtA has been previously shown to be repressed during 130b growth in media containing higher amounts of iron [35]. Values are means and standard deviations from three-independent experiments. (TIF) [file pone.0046209.s002.tif]

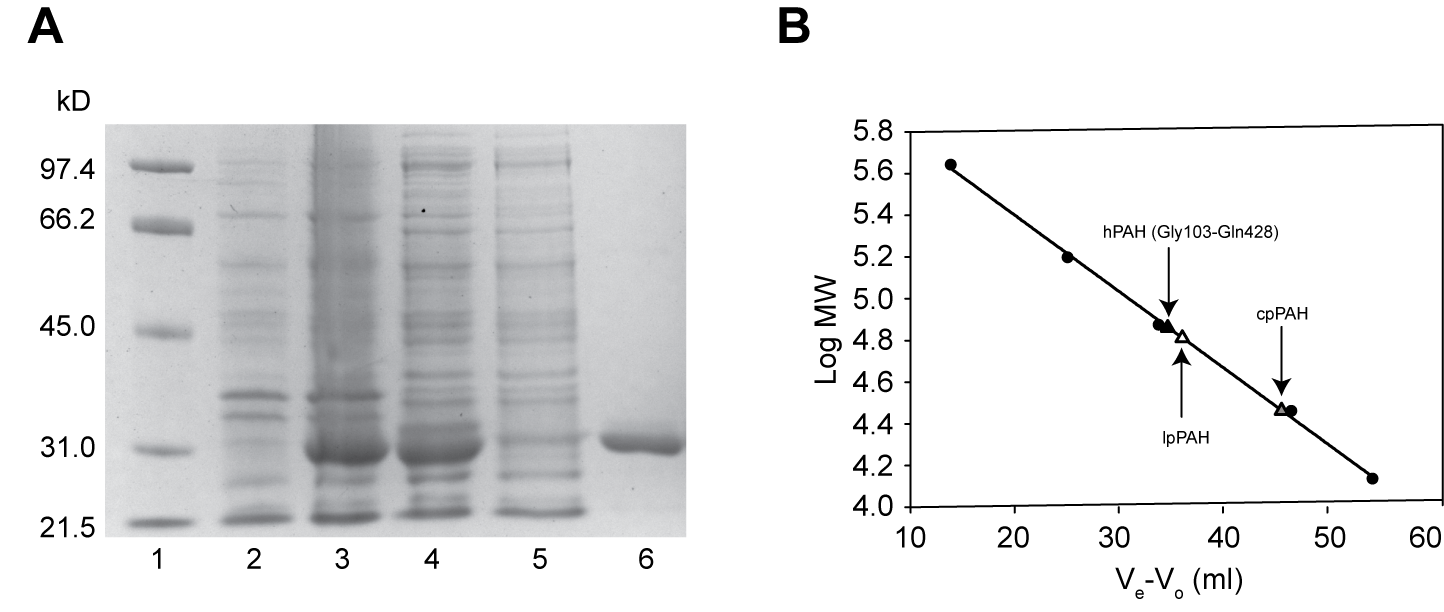

Supplement: Figure S3 — Expression, purification and size determination of recombinant lpPAH. A) SDS-PAGE analysis of the overexpression and purification of lpPAH-(His)6 by affinity chromatography. Lane 1, low molecular weight markers; lane 2, uninduced culture; lane 3, induced culture; lane 4, crude extract (soluble fraction); lane 5, flow-through from Talon column; lane 6, eluted protein fraction from Talon column (purified lpPAH). B) Calibration curve for the determination of protein molecular weight by size exclusion chromatography with a HiLoad Superdex column (1.6 cm×60 cm) at a flow rate of 1 ml/min. The black circles represent the positions for the following proteins used for calibration: ferritin (440 kD), aldolase (158 kD), conalbumin (75 kD), carbonic anhydrase (29 kD) and ribonuclease A (13.7 kD). The position of hPAH(Glyl03-Gln428) (dimer of 70 kDa); cpPAH (monomer of 30.7 kDa) and lpPAH (estimated from this calibration to be a dimer of 64.5 kDa) are also shown. Ve, elution volume; Vo, void volume. (TIF) [file pone.0046209.s003.tif]
